# Mechanosynthesis of the Whole Y_1−x_Bi_x_Mn_1−x_Fe_x_O_3_ Perovskite System: Structural Characterization and Study of Phase Transitions

**DOI:** 10.3390/ma12091515

**Published:** 2019-05-09

**Authors:** Jose Ángel Quintana-Cilleruelo, Vignaswaran K. Veerapandiyan, Marco Deluca, Miguel Algueró, Alicia Castro

**Affiliations:** 1 Instituto de Ciencia de Materiales de Madrid, CSIC, Cantoblanco, 28049 Madrid, Spain; malguero@icmm.csic.es (M.A.); acastro@icmm.csic.es (A.C.); 2 Materials Center Leoben Forschung GmbH, Roseggerstr. 12, 8700 Leoben, Austria; Vignaswaran.Veerapandiyan@mcl.at (V.K.V.); marco.deluca@mcl.at (M.D.)

**Keywords:** mechanochemical processing, YMnO_3_/BiFeO_3_, oxide materials, phase transition, ferroelectric, antiferromagnetic

## Abstract

Perovskite BiFeO_3_ and YMnO_3_ are both multiferroic materials with distinctive magnetoelectric coupling phenomena. Owing to this, the Y_1−x_Bi_x_ Mn_1−x_Fe_x_O_3_ solid solution seems to be a promising system, though poorly studied. This is due to the metastable nature of the orthorhombic perovskite phase of YMnO_3_ at ambient pressure, and to the complexity of obtaining pure rhombohedral phases for BiFeO_3_-rich compositions. In this work, nanocrystalline powders across the whole perovskite system were prepared for the first time by mechanosynthesis in a high-energy planetary mill, avoiding high pressure and temperature routes. Thermal decomposition temperatures were determined, and structural characterization was carried out by X-ray powder diffraction and Raman spectroscopy on thermally treated samples of enhanced crystallinity. Two polymorphic phases with orthorhombic Pnma and rhombohedral R3c h symmetries, and their coexistence over a wide compositional range were found. A gradual evolution of the lattice parameters with the composition was revealed for both phases, which suggests the existence of two continuous solid solutions. Following bibliographic data for BiFeO_3_, first order ferroic phase transitions were located by differential thermal analysis in compositions with x ≥ 0.9. Furthermore, an orthorhombic-rhombohedral structural evolution across the ferroelectric transition was characterized with temperature-dependent X-ray diffraction.

## 1. Introduction

Multiferroic materials are characterized by the coexistence of at least two of the three ferroic orders (ferroelectric, ferromagnetic, and ferroelastic). Most topical systems are those that involve the coupling of ferroelectricity and ferromagnetism or antiferromagnetism. These materials are interesting due to their prospective application in potentially disruptive technologies based on their magnetoelectric coupling characteristics, such as electrically tunable microwave magnetic components, spintronic devices, or electrically writable/magnetically readable random access memories [1,2]. However, only a few materials are both ferroelectric and ferromagnetic and there is no material with robust magnetoelectric coupling at room temperature.

Perovskite-type orthorhombic manganese oxides (o-RMnO_3_, where R refers to rare earth) is a group of multiferroic materials. In these materials, ferroelectric polarization is directly induced by its magnetic order, and by definition are called spin-driven ferroelectrics [3]. This crystal structure is only stable for R species with a large ionic radius, so that when the radius of the rare-earth ion is small enough (r_R_ < r Dy^3+^) [4], the orthorhombic phase becomes metastable and a hexagonal phase is formed. In these cases, the orthorhombic phase can only be obtained by non-conventional methods, like soft chemistry [5], high-pressure techniques [6], or mechanosynthesis, only the last of which ensures the avoidance of secondary phases [7]. The low-density hexagonal phase is promoted by the Jahn–Teller effect on the local octahedral environment of the Mn^3+^ cation. Mn^3+^ ions adopt a high spin configuration, t_2g_^3^e_g_^1^, due to the strong Hund’s rule coupling. The distortion produced by lengthening two of the Mn–O trans bonds lowers the energy of the occupied 3d_z_^2^ orbitals with respect to the empty 3d_x_^2^_−y_^2^ orbitals. As a result, the filled 3d_z_^2^ orbitals form a zigzag pattern in the x-y plane, which leads to an expansion of the *a* and *c* unit cell parameters [7], and a subsequent decrease in the density of the unit cell. YMnO_3_ is a good example of this phenomenon. The stable phase at ambient conditions is the low-density hexagonal structure. This so-called geometric ferroelectric orders into an A-type antiferromagnetic phase at 70 K. YMnO_3_ can also exist in the metastable high-density orthorhombic structure that shows an E-type antiferromagnetic order below 40 K, and magnetically driven ferroelectricity.

BiFeO_3_ is the most topical multiferroic material, and one of the rare examples that show ferroelectricity and antiferromagnetism at room temperature. With a rhombohedrically distorted perovskite structure, its ferroelectricity is driven by the lone-pair electron stereoactivity, based on the spatial asymmetry created by the anisotropic distribution of the 6s^2^ unbounded valence electrons of Bi^3+^, which induces a local dipole [8]. A paraelectric phase appears above 1103 K with an orthorhombic structure (S.G.: Pnma, No 62), isostructural with the metastable o-YMnO_3_. Regarding its magnetic order, this has been described as a G-type antiferromagnetic phase, where each spin moment of the Fe^3+^ is surrounded by six nearest Fe^3+^ neighbors with antiparallel moments. A spiral modulation is superimposed, which can be destroyed by decreasing the grain size below the characteristic length of the spin cycloid (62 nm) or by chemical substitutions, among others [9]. Spin canting and weak ferromagnetism are then uncovered [10]. The synthesis of pure BiFeO_3_ presents difficulties, and Bi_2_Fe_4_O_9_ or Bi_25_FeO_39_ phases commonly appear due to the poor stability of BiFeO_3_ at high temperatures [9,11,12,13].

It is well-known that ferroelectric solid solutions between two perovskite systems with different structural symmetries can present a structural transition region in their phase diagrams with the coexistence of polymorphs in a range of compositions, known as a morphotropic phase boundary (MPB), where the electrical and electromechanical properties are improved [14]. This property enhancement is associated with a polarization rotation mechanism in anisotropically flattened free energy profiles, or alternatively with a polarization extension mechanism near Tc. Polarization extension is relevant at the temperature-driven ferroelectric-paraelectric transition, with the disadvantage of a poor temperature stability of the enhancement. This handicap can be avoided in solid solutions that exhibit polar-nonpolar MPB [15,16]. The anticipated compositionally induced structural change across the binary system, (1−x)YMnO_3_–xBiFeO_3_, is in accordance with a hypothetical polar–non polar MPB, and the multiferroic nature of the end systems suggests the possibility of obtaining an enhancement of the magnetoelectric properties at the MPB [17]. This enhancement might be further increased by the expected decrease of the T_c_ value from that of BiFeO_3_ down to values close to ambient temperature.

Mechanochemical methods allow most typical perovskite oxides to be obtained at room temperature [18,19,20] and, therefore, to avoid the formation of secondary phases as in the case of BiFeO_3_ [21]. This method also promotes the synthesis of high-density phases under the high point pressures of the order of GPa exerted during the milling, without the application of external pressure [22,23], as required to obtain metastable o-YMnO_3_ [7]. As a result, more dense polymorphs are stabilized at room temperature [23]. To the best of our knowledge, only a few reports dealing with the synthesis of selected compositions of (1−x)YMnO_3_–xBiFeO_3_ have been published [24,25,26], and only one of them addressed the synthesis of the whole binary system. However, the synthesis of all the compositions was not achieved in that work, but secondary phases appeared in most of them [26]. In this paper, the successful synthesis of the whole (1−x)YMnO_3_–xBiFeO_3_ perovskite system is reported for the first time by mechanochemical methods. All pure phases of the binary system were obtained, due to the stabilization of high-pressure metastable phases. A structural study throughout the entire system has been carried out using X-ray diffraction and Raman spectroscopy techniques, and phase evolution has been described. A wide region of coexistence between orthorhombic and rhombohedral perovskite structures has been found. Additionally, the existence of ferroic phase transitions has been investigated.

## 2. Materials and Methods

Thirteen compositions across the system of (1−x)YMnO_3_–xBiFeO_3_ were prepared from a stoichiometric mixture of analytical grade Bi_2_O_3_, Fe_2_O_3_, Y_2_O_3_, and Mn_2_O_3_: x = 0, 0.1, 0.2, 0.3, 0.4, 0.5, 0.6, 0.7, 0.8, 0.9, 0.925, 0.95, and 1. Initial mixtures were thoroughly ground in an agate mortar until a homogeneous mixture was formed, and about 10 g of this mixture were mechanically treated in a planetary mill (Fritsch Pulverisette 4) using a tungsten carbide vessel, with a volume of 250 cm^3^ and seven tungsten carbide balls, 20 mm in diameter and 63 g in weight each. Milling was carried out using a sequence of 30 min milling and 10 min resting to prevent overheating of the system. The grinding vessel was rotated at 300 rpm with a translation/rotation ratio of 0.82, during increasing times, under ambient atmosphere, until a perovskite single-phase oxide was obtained. Phase evolution during mechanical treatment was followed by X-ray powder diffraction, with a Bruker AXS D8 Advance diffractometer (Bruker, Karlsruhe, Germany), between 12° and 60° (2θ), with 2θ increments of 0.05 and a counting time of 0.2 s per step. The Cu-Kα doublet (λ = 1.5418 Å) was used in the experiments. The morphology of the samples was examined by field emission scanning electron microscopy, performed with a Philips XL 30 S-FEG FE-SEM (Philips, Eindhoven, The Netherlands). The resolution of the equipment was 3.5 nm, using ultrahigh vacuum and a work range of 1 to 30 kV with a secondary electron detector. All samples were prepared with a sputter Quorum metallization Q150T-S (Quorum Technologies, Laughton, UK).

The mechanosynthesized powders were thermally treated at increasing temperatures for 1 h, from 400 °C up to the decomposition temperature. The samples for the structural characterization were prepared by thermal annealing to temperatures until before the onset of thermal decomposition. Crystallographic evolution across the system was studied by X-ray powder diffraction, with the same equipment used to monitor the synthesis. For determination of the cell parameters, diffraction patterns were performed between 20° and 60° (2θ), with 2θ increments of 0.02 and a counting time of 2.7 s per step. Refinements were performed using a least-square method (CELREF) [27].

Raman measurements were performed with LabRAM and LabRAM HR800 (Horiba Jobin Yvon, Villeneuve d’Ascq, France) spectrometers using a 532 and 633 nm laser excitation, respectively. The laser beam spot has a diameter of ~1 µm on the specimen surface. Spectra were collected in backscattering geometry with an 1800 gr/mm grating and a Peltier-cooled charged coupled device (CCD) camera, allowing a spectral resolution of at least 1.5 ± 0.1 cm^−1^/pixel for the investigated range. The measured spectra were deconvoluted with a sum of the Gaussian–Lorentzian peak functions in a commercial software environment (Labspec 4.02; Horiba Jobin Yvon).

BiFeO_3_ ferroic phase transitions can be located by differential thermal analysis (DTA) [9,28]. According to this, possible ferroic phase transitions were identified by DTA, carried out with two simultaneous thermal analysis equipment, ATD/DSC/TG model Q600 TA Instruments (TA Instruments, New Castle, EEUU), between room temperature and 850 °C, with heating and cooling rates of 10 °C·min^−1^. The nature of the phase transitions was further investigated by temperature-dependent X-ray diffraction, which was performed with a Panalytical X’Pert PRO θ/θ diffractometer (Panalytical, Almelo, The Netherlands) equipped with an HTK 1200N high-temperature oven chamber (Anton Paar, Graz, Austria), between room temperature and 810 °C with a heating rate of 10 °C·min^−1^ and a stabilization time of 10 min before the measurement. Diffraction patterns were performed between 20° and 60° (2θ), with 2θ increments of 0.05 and a counting time of 2 s per step. The Cu-Kα doublet (λ = 1.5418 Å) was used in the experiments.

## 3. Results and Discussion

### 3.1. Mechanosynthesis

**Figure 1:**
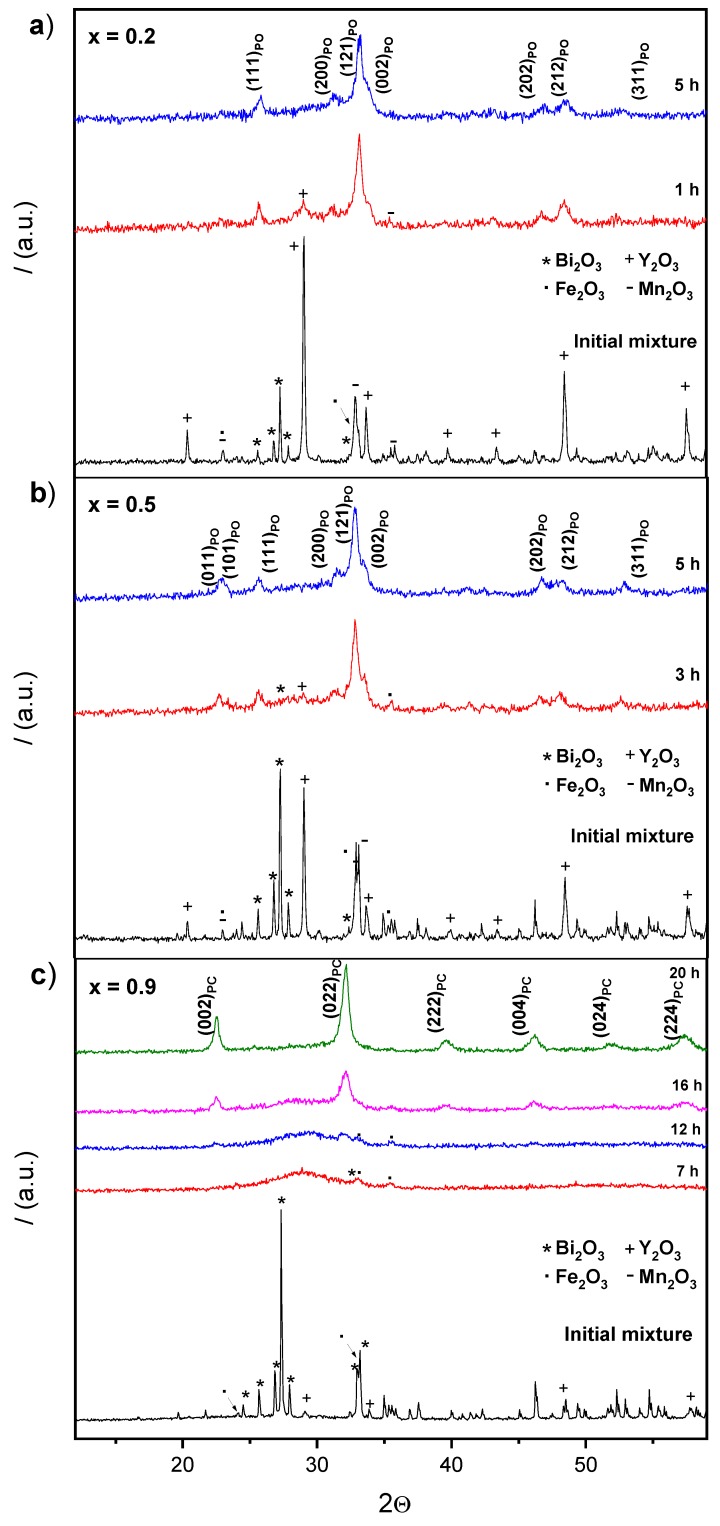
Evolution with the milling time of the stoichiometric mixture of reactants corresponding to Y_1−x_Bi_x_Mn_1−x_Fe_x_O_3_ for compositions: (**a**) x = 0.2; (**b**) x = 0.5; and (**c**) x = 0.9 (PC—Pseudocubic Perovskite; PO—Pseudo-orthorhombic Perovskite).

In this work, the stoichiometric mixtures of reactants were mechanically treated in a high energy planetary mill as a new route to synthesize the whole (1−x)YMnO_3_–xBiFeO_3_ binary system. As a procedure to determine the minimum milling time necessary to complete the mechanosynthesis (reaction time), X-ray diffraction (XRD) was used to study the evolution of the reaction, and the time required for its completion was defined as that when the X-ray diffractograms showed no amorphous or starting products, as well as the absence of secondary phases. As an example, Figure 1 shows the diffraction patterns of the initial mixture of starting oxides for the x = 0.2, 0.5, and 0.9 compositions and the products after increasing milling. Different times were required to complete the mechanosynthesis for different compositions, and all values are reported in Table 1. Note for compositions, x = 0.2 and x = 0.5, the reaction evolves very fast, and that the perovskite is already present after only 1 h. Reaction was slower for composition x = 0.9 (Figure 1c), which allowed a description of the process to be made. Namely, a decrease of the crystallinity of the reactants until amorphization initially took place (7 h), and a subsequent mechanochemical reaction in the activated mixture led to the formation of a nanocrystalline perovskite-phase after 16 h of milling. Single phase perovskite-oxide was achieved after 20 h when the diffraction pattern did not exhibit an amorphous phase.

**Figure 2:**
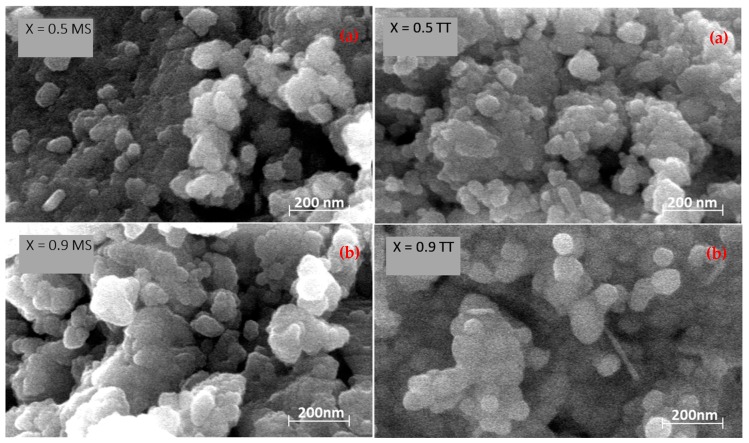
Scanning electron micrographs of powders mechanosynthesized (MS), and thermally treated (TT) at the maximum temperature before decomposition for (**a**) Y_0.5_Bi_0.5_Mn_0.5_Fe_0.5_O_3_ and (**b**) Y_0.1_Bi_0.9_Mn_0.1_Fe_0.9_O_3_.

Regarding the obtained phases, a structural evolution with composition was found, from a pseudo-orthorhombic perovskite for low x values (e.g., Figure 1a), to a pseudo-cubic perovskite for high x values (e.g., Figure 1c). Specific symmetry in the latter case could not be identified from the XRD patterns of the mechanosynthesized oxides due to peak broadening, reflecting the small crystal size. This was revealed by the scanning electron microscopy studies: A micrograph of the mechanosynthesized powder with x = 0.5 and x = 0.9 is shown in Figure 2a, where submicron sized agglomerates of small particles with a particle size between 20 and 25 nm are observed. Significant differences in particle size with composition were not found.

In order to increase the crystallinity of the nanocrystalline powders for the structural characterization, thermal treatments were carried out at the highest temperature before the perovskite decomposition (cf. Figure 2b). No secondary phases developed during the intermediate treatments, which confirmed the completion of the mechanosynthesis, and demonstrated that this technique is suitable to overcome the problems to isolate single phases in BiFeO_3_-rich compositions, previously found with other synthesis methods [24,25]. The maximum temperature possible before decomposition was found to be composition dependent (cf. values in Table 1). A limited particle growth took place during the thermal treatments, resulting in sizes between 65 and 80 nm (Figure 2b). The particle size then remained in the nanoscale. 

### 3.2. Structural Characterization

#### 3.2.1. XRD Studies

**Figure 3:**
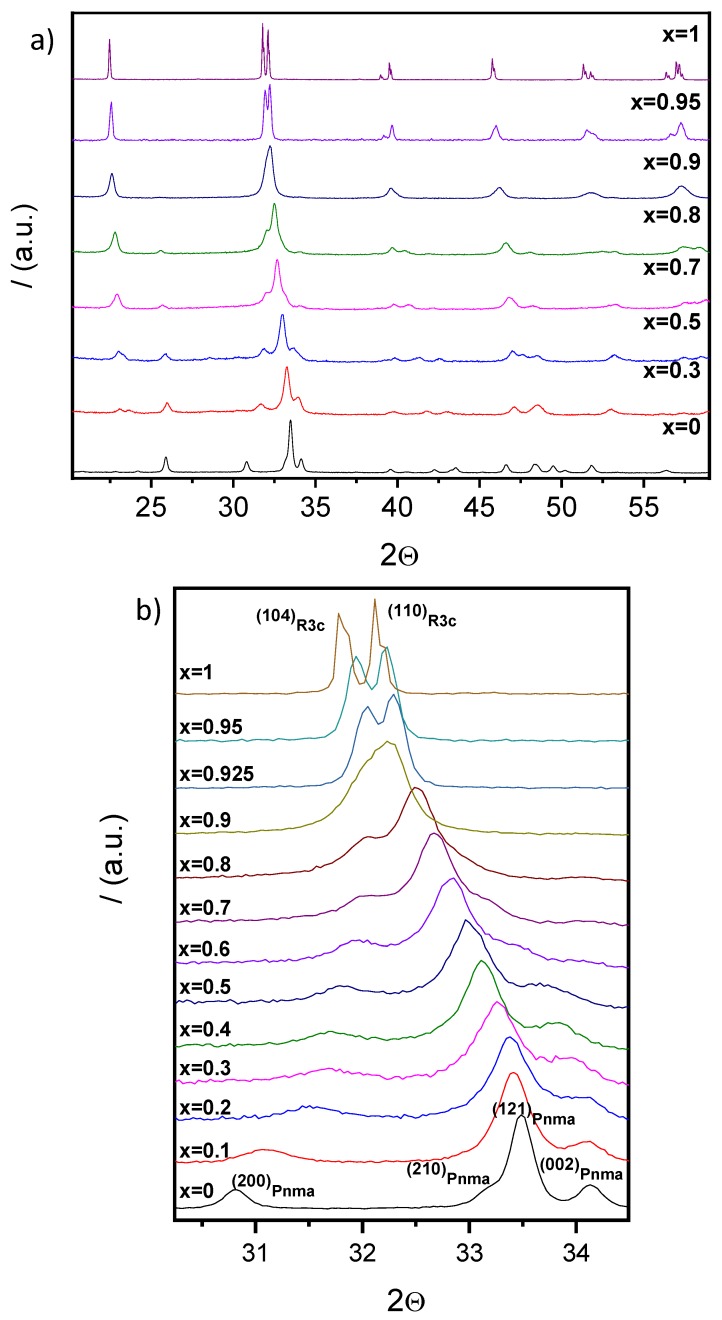
XDR patterns of the (1−x)YMnO_3_–xBiFeO_3_ phases obtained by mechanosynthesis and subsequently thermally treated at the maximum temperature before decomposition (**a**) for selected compositions and (**b**) for all compositions and selected 2θ angles.

Distinctive splittings of the perovskite diffraction peaks developed as crystallinity increased, as illustrated in Figure 3a, where the XRD patterns of the mechanosynthesized and thermally treated samples are given for selected compositions. An evolution of symmetry is thus apparent. All patterns can be indexed to Pnma (YMnO_3_ S.G. No 62), R3c h (BiFeO_3_ S.G. No. 161), or a mixture of both. Indeed, three regions can be defined: (I) x < 0.5, where only the orthorhombic Pnma symmetry can be identified; (II) 0.5 ≤ x ≤ 0.9, where the increment of intensity of a signal at 2θ ≈ 23° is related to the appearance of the rhombohedral R3c h phase, showing the coexistence of both symmetries; and (III) x > 0.9, where only the rhombohedral R3c h phase is detected. Note the gradual displacement of the diffraction lines with the composition across the system, as shown in Figure 3b for the crystallographic planes (121)_Pnma_ and (104)_R3ch_–(110)_R3ch_ from the orthorhombic and rhombohedral structures, respectively. This strongly suggests the correct formation of the perovskite solid solutions across the whole binary system.

**Figure 4:**
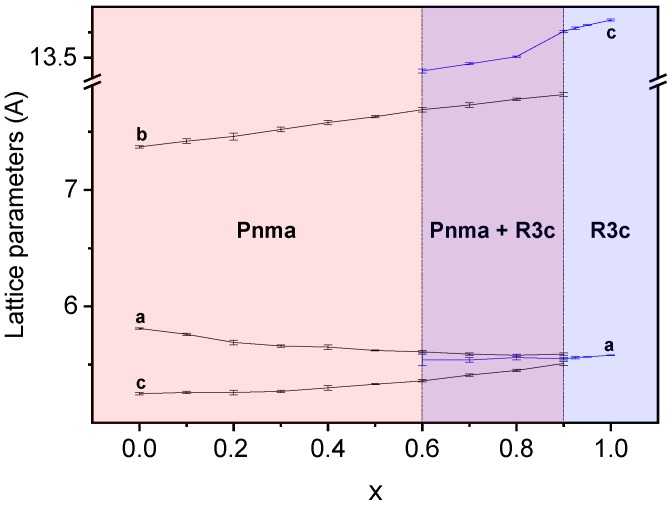
Unit-cell parameters with corresponding errors for the orthorhombic Pnma (S. G. No. 62) and rhombohedral R3c h (S. G. No. 161) phases in the (1−x)YMnO_3_–xBiFeO_3_ system.

Lattice parameters as a function of the composition were obtained by the least-squares method for both perovskite phases. Results are summarized in Figure 4 and Table 1. Although coexistence of the orthorhombic Pnma (S.G. No. 62) and rhombohedral R3c h (S.G. No. 161) phases was observed in the range of 0.5 ≤ x ≤ 0.9, only one peak at 2θ ≈ 23° can be assigned to the rhombohedral phase in the diffraction pattern of the composition, x = 0.5, due to the low intensity and overlapping of signals. As a result, parameters for the R3c h phase could not be obtained for this composition. Trends for both polymorphs are given in Figure 4.

In the case of the orthorhombic phase, lattice parameters of the Pnma symmetry have been obtained for compositions from x = 0 to x = 0.9. The tendency of lattice parameter *a* is to decrease along the binary system, while that of lattice parameter *c* is to increase, so that they become equal within error for x = 0.9. The lattice parameter, *b*, steadily increases with a final increment of almost 0.5 Å. These changes in the Pnma lattice parameters result in an increment of the volume of the orthorhombic unit cell, as will be discussed later. In the case of the rhombohedral R3c h phase, the calculation of lattice parameters is possible from x = 0.6 to x = 1 as stated in the previous paragraph. Lattice parameter *c* increases with x, while lattice parameter *a* maintains a constant value.

**Figure 5:**
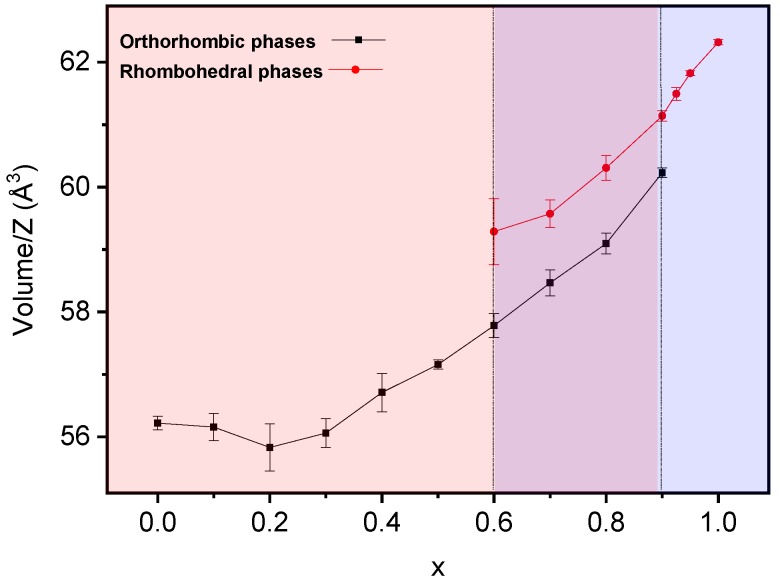
Unit-cell volume per formula unit with corresponding errors for the orthorhombic Pnma (S. G. No. 62) and rhombohedral R3c h (S. G. No. 161) phases in the (1−x)YMnO_3_–xBiFeO_3_ system.

The evolution of the unit cell volume across the binary system is shown in Figure 5. Note that for compositions from x = 0 to x = 0.2, the Pnma unit cell volume slightly decreases. This could be due to the opposite effects produced by the specific cations substituting in “A” and “B” sites of the perovskite. On the one hand, an increment of the unit cell volume with x is expected in terms of the ionic radii of Y^3+^ and Bi^3+^ (1.019 Å and 1.17 Å, respectively [29]). On the other hand, the substitution of Mn^3+^ for Fe^3+^ in the “B” site implies the progressive decrease of the Jahn–Teller effect and the subsequent diminution of the unit cell volume, as it was explained in [7].

It follows that the Jahn–Teller effect mostly determines the evolution of the unit cell volume until composition x = 0.2. From this composition, the effect of substitution in the “A” site dominates over Mn^3+^ substitution, and an expansion of the unit cell results (Figure 5). The rhombohedral R3c h unit cell volume increases with composition, a trend in good agreement with Bi^3+^ substituting for Y^3+^ in the “A” site of the perovskite.

#### 3.2.2. Raman Studies

**Figure 6:**
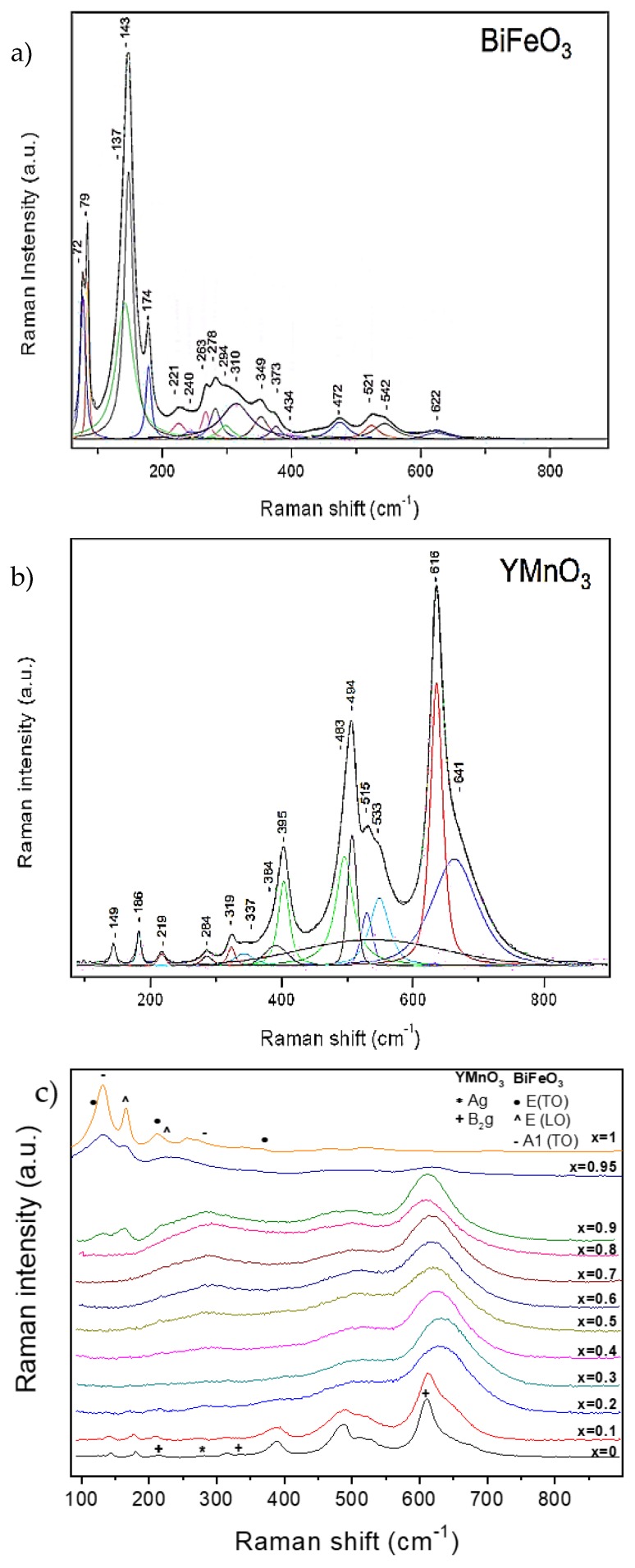
Raman spectrum of the (**a**) BiFeO_3_, (**b**) YMnO_3_, and (**c**) (1−x)YMnO_3_–xBiFeO_3_ phases.

The room temperature Raman spectra of the thermally treated powders are shown in Figure 6. Raman results confirmed the phase evolution across the system, as well as the phase coexistence across a wide compositional region. BiFeO_3_ and YMnO_3_ are presented separately as end members whose crystal structure and lattice dynamics are known (Figure 6a,b, respectively).

Raman modes of BiFeO_3_ and YMnO_3_ have been assigned in Figure 6a,b, according to the literature [30,31,32], and are also listed in Table 2 and Table 3. Note that it is not possible to identify all modes of both oxides due to the use of a single scattering configuration and the dispersion of phonon wavevectors characteristic of measurements on powdered samples. Nevertheless, all observed modes are in accordance with those reported in the literature. For BiFeO_3_, and out of the 26 phonon modes reported [30], 18 optic frequencies have been identified. These are 13 transversal optic phonon frequencies [9E(TO) + 4A_1_(TO)], and 5 longitudinal optic phonon frequencies [5E(LO)]. In the case of YMnO_3_, vibrational modes of motions within the x-z plane (A_g_ and B_2g_ modes) and B_1g_ mode assigned to in-phase x-rotation of oxygens along the y-axis have been observed [31].

As compared with those of the reference oxides, YMnO_3_ and BiFeO_3_, the Raman spectra of the phases of Y_1−x_Bi_x_Mn_1−x_Fe_x_O_3_ (0.1 ≤ x ≤ 0.95) appear much broader. A possible reason for this phenomenon is that A-site and B-site substitution produces site disorder, short-range lattice distortion, and even chemically ordered regions. All these aspects relax the first-order Raman selection rules [33] and thus produce a broader spectral appearance. An alternative mechanism for broadening has been proposed by Iliev et al. [34], and explains that the broad spectra are indicators of a smeared partial phonon density-of-states (PDOS) referred to oxygen vibrations. Indeed, the coexistence at the B-site of cations whose octahedral oxygen environment is affected by the Jahn–Teller effect (B-site occupied by Mn^3+^) and cations that do not produce this phenomenon (B-site occupied by Fe^3+^) causes a strong disorder of the oxygen sublattice, which produces a smeared partial phonon density-of-states (PDOS). The consequent disappearance of translational symmetry activates Raman-forbidden oxygen vibrations, corresponding to off-center phonon modes in the pure YMnO_3_.

**Figure 7:**
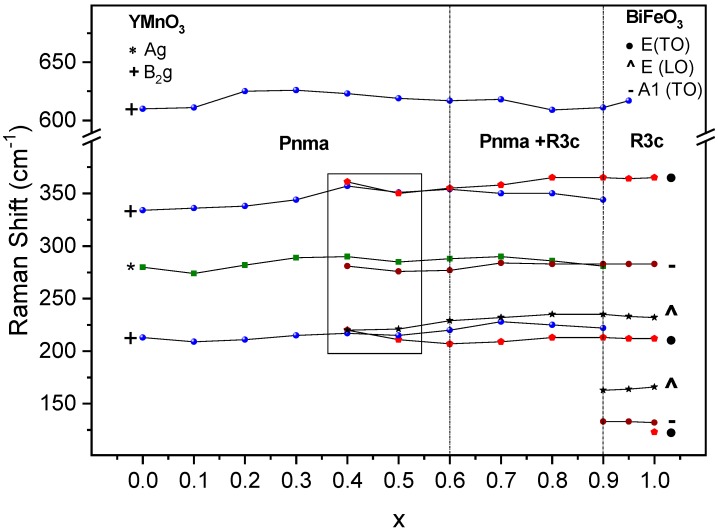
Position of selected Raman modes throughout the (1−x)YMnO_3_–xBiFeO_3_ system.

Regarding specific phases, it is possible to differentiate three different regions according to the changes in the vibrating modes: Starting from YMnO_3_, the spectra are consistent with the orthorhombic single phase up to x = 0.3, for which all orthorhombic modes can be identified—yet highly broadened as discussed. The first signature modes of the rhombohedral phase are found starting from x = 0.4, initially in coexistence with the orthorhombic ones, and are isolated only above 0.9. The strong modification of the spectrum from x = 0.9 to 0.95, as shown in Figure 6c, is very remarkable, and points at the end of phase coexistence, with the attainment of a rhombohedral single phase region. The evolution of selected modes with the composition is given in Figure 7 along with the phase evolution detected by XRD (dashed lines). In the range of 200 to 350 cm^−1^, it is not possible to distinguish between the orthorhombic A_g_ mode and the rhombohedral vibrational E(TO) and E(LO) modes for compositions of 0.4 < x < 0.6, although the broad spectral appearance suggests phase coexistence from x = 0.4 onwards.

Regions defined with Raman compare well with those defined with XRD, although the width of the coexistence region is expanded down to x = 0.4. Note that these techniques have different spatial resolutions, and that Raman is sensitive to very short-ranged structural orders that are beyond XRD capabilities. It can thus be argued that phase coexistence appears on the short range already at x = 0.4 and develops to the long range by x = 0.6.

### 3.3. Phase Transitions and Phase Stability

First order ferroelectric and antiferromagnetic phase transitions of BiFeO_3_ [9] have associated characteristic thermal effects that can be observed with differential thermal analysis (DTA) [28]. Therefore, measurements were carried out for the whole (1−x)YMnO_3_–xBiFeO_3_ system. Specifically, distinctive thermal effects that could be associated with ferroic transitions were only found for compositions of 0.9 ≤ x ≤ 1. DTA measurements consisted of two consecutive heating-cooling cycles reaching temperatures up to the temperature of the crystallization treatment, starting from mechanosynthesized phases.

**Figure 8:**
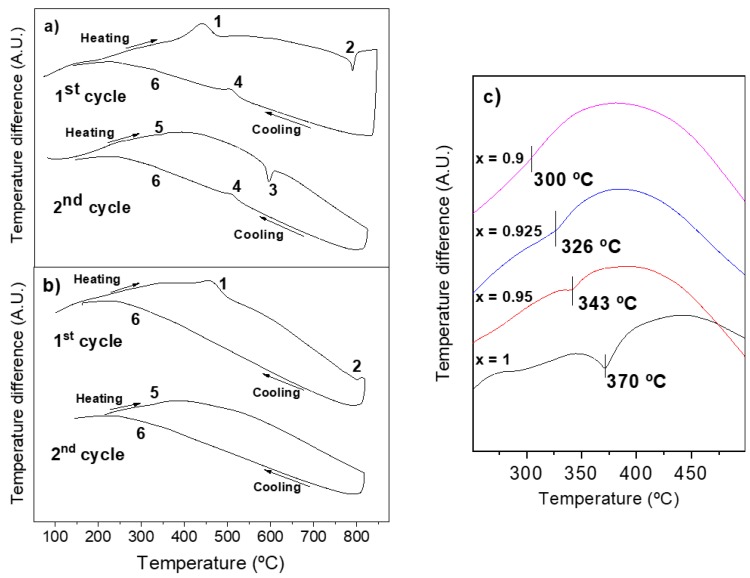
DTA curves of as-mechanosynthesized (1−x)YMnO_3_–xBiFeO_3_ powders during two successive heating-cooling cycles: (**a**) x = 0.95, and (**b**) x = 0.925. Main thermal effects are labelled as 1–6. (**c**) Evolution of effect 5 during the second cycle on heating for compositions with 0.9 ≤ x ≤ 1.

Figure 8a,b show the first and second heating-cooling cycles for compositions of x = 0.95 and 0.925, respectively. Equivalent thermal responses were found for x = 1 (to x = 0.95) and x = 0.9 (to x = 0.925). The first cycle of all compositions shows an initial exothermic effect (labelled as 1 in the figure). This thermal event is irreversible (not observed in the second cycle), and takes place at temperatures ranging from 410 °C to 460 °C. It is a common effect of nanocrystalline perovskite oxides obtained by mechanosynthesis, which has been attributed to the crystallization of the powder [21]. A second effect, this time endothermic and also irreversible, is observed at high temperatures in the range 790–800 °C (labelled as 2). Its origin is not clear, but it seems to be associated somehow with the ferroelectric transition, as it will be explained later. On subsequent cooling, two successive exothermic effects (labelled as 4 and 6) are observed for compositions, x = 1 (at 805 °C and 363 °C) and x = 0.95 (504 °C and 335 °C), while a weak single effect is detected for x = 0.925 (318 °C) and x = 0.9 (295 °C). Note that the effect labelled as 6 can hardly be discerned in Figure 8 due to the scale used. Nonetheless, their presence and temperature values were unambiguously determined with the first derivative of the cooling curve. During the following second cycle on heating, two new endothermic peaks labelled as 5 and 3 are observed for x = 1 (at 370 °C and 834 °C) and x = 0.95 (at 343 °C and 594 °C), while only effect 5 is detected for x = 0.925 (326 °C) and x = 0.9 (300 °C). All events observed in the first cycle are then reproduced during final cooling.

According to the bibliography [9,28], the Curie temperatures, T_c_, of the ferro-para-ferroelectric phase transition of BiFeO_3_ are 825 to 830 °C on heating and 800 to 810 °C on cooling. These temperatures are those of events 3 (830 °C) and 4 (803 °C) for composition x = 1, so it is reasonable to associate these thermal effects with the ferroelectric transition. Therefore, the analogous events at 594 and 504 °C for x = 0.95 are most probably associated with this transition that has been shifted towards low temperatures in the solid solution. Note that the thermal effect associated with the ferroelectric-paraelectric transition on heating (event 3) for composition x = 0.95 is observed in the second cycle, but not in first cycle. Actually, additional experiments have shown that events 3 and 4 do not appear unless the first cycle reaches the temperature of the irreversible effect labelled as 2. This suggests the ferroelectric phase to be locked in the as-mechanosynthesized powder, so that the first ferroelectric-paraelectric phase transition takes place at an anomalous high temperature. The mechanism by which this appears is not clear at this stage. Thermal events related with T_c_ are not observed in the DTA curves of the compositions, x = 0.925 and x = 0.9 (see Figure 8b), which might indicate the disappearance of the phase transition, but also a change in the phase transition character from first-order to second-order, or even a decrease of the latent heat.

**Figure 9:**
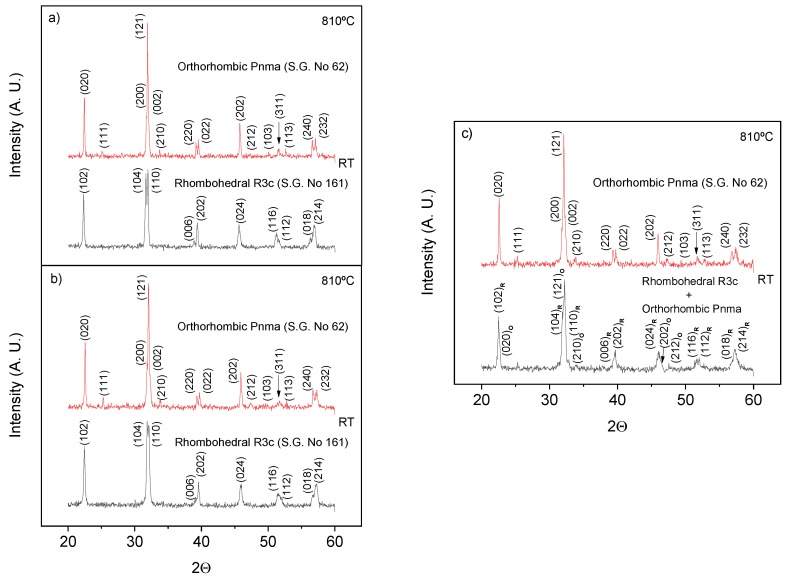
X-ray diffraction patterns at room temperature and 810 °C for (1−x)YMnO_3_–xBiFeO_3_ with (**a**) x = 0.95, (**b**) x = 0.925, and (**c**) x = 0.9.

In order to confirm the association of events 3 and 4 with the ferroelectric phase transition, temperature-dependent X-ray diffraction experiments were carried out. XRD patterns at room temperature and 810 °C are presented in Figure 9a for x = 0.95. A clear structural change from rhombohedral R3c h (S.G. No. 161) to orthorhombic Pnma (S.G. No. 62) was found, as expected to occur across the ferroelectric transition [9]. Moreover, measurements were also carried out for x = 0.925 (Figure 9b) and 0.9 (Figure 9c). An analogous evolution to that described for x = 0.95 was found for the former case, which indicates the occurrence of the phase transition, even if thermal effects were not observed. For x = 0.9, the room temperature X-ray diffraction pattern corresponds to the coexistence of two structural phases as stated in Section 3.1, while a single orthorhombic phase is found at high temperatures.

DTA results show a second couple of reversible thermal effects at intermediate temperatures, labelled as 5 and 6 in Figure 8a,b. They take place at 370 °C in the case of BiFeO_3_, which are the temperatures of the antiferro-paramagnetic phase transition (T_N_), according to the literature [9]. They are observed for all compositions down to 0.9 and allow T_N_ to be defined as a function of x. The trend is shown in Figure 8c. Note that the Neel temperature shifts towards lower temperatures in agreement with the literature [23], while the intensity of the thermal effect decreases as the composition approaches x = 0.9.

### 3.4. Considerations about Mechanosynthesis and Thermal Stability

The results shown here demonstrate that mechanical activation in high-energy planetary mills is a suitable technique to achieve the mechanosynthesis of the whole Y_1−x_Bi_x_Mn_1−x_Fe_x_O_3_ perovskite system. A distinctive trend of the required reaction time with compositions has been found, which can be related with the phase evolution revealed by the structural characterization.

In the 0 ≤ x ≤ 0.4 range, where an orthorhombic perovskite single phase exists, reaction times decrease from 8 h down to 3 h. This trend correlates with the perovskite tolerance factor, *t*, defined as:

(1) $$t\;=\;\frac{r_{A}+r_{O}}{\sqrt{2}\left(r_{B}+r_{O}\right)}$$

where *r*_A_, *r*_B_, and *r*_O_ are the ionic radii of the A and B cations and O^−2^ anion, respectively [35]. The substitution of Bi^3^ for Y^3+^ produces a change in the ionic radius of the perovskite A-site (1.019 Å for Y^3+^ as compared with 1.17 Å for Bi^3+^ [29]), which results in a continuous increase of the tolerance factor, from 0.84 for x = 0 up to 0.87 for x = 0.4. Stable perovskite structures can be formed in the range of tolerance factor, 0.77 < *t* < 0.99 [36]. Therefore, the reduction of the milling time with x might reflect the increasing stability of the perovskite structure. In the 0.9 < x ≤ 1 range, where a rhombohedral perovskite single phase exists, the correlation of time with perovskite tolerance factor holds, so that the required mechanical reaction time decreases as x increases from 20 h (x = 0.9) to 14 h (x = 1). Note that required milling times for the orthorhombic phase are significantly shorter than those for the rhombohedral one. This explains the evolution of the milling time in the phase coexistence region (i.e., milling time increase with x for 0.4 < x ≤ 0.9), which basically reflects the increasing percentage of the rhombohedral phase with x.

A distinctive trend of the maximum temperatures before decomposition across the system has also been found (cf. Table 1). Thermal stability of the pure orthorhombic perovskite phase decreases when x increases, which cannot be explained with the tolerance factor (which increases) or with the disappearance of the Jahn–Teller effect. Therefore, an alternative explanation is required that might refer to the modification of the phase evolution during heating. Actually, these phases do not directly turn into a hexagonal phase like YMnO_3_ does, but they decompose into a new secondary phase, likely consisting of bismuth and iron mainly, and a hexagonal phase. Decomposition products may change with composition, which might explain the thermal stability of the perovskite system. In the range of coexistence of the orthorhombic and rhombohedral phases and the region of a pure rhombohedral phase, an increase of thermal stability is found, in good agreement with the increasing tolerance factor.

## 4. Conclusions

Perovskite single phase nanocrystalline powders were obtained for the first time across the whole (1−x)YMnO_3_–xBiFeO_3_ binary system by mechanosynthesis in a high-energy planetary mill with tungsten carbide grinding media. A characteristic evolution of the required reaction time with the composition was found, which relates with the perovskite tolerance factor, and with the specific polymorphic phase synthesized, either orthorhombic or rhombohedral. Powders with enhanced crystallinity for structural characterization were prepared by subsequent thermal treatments. X-ray diffraction and Raman spectroscopy have allowed the specific perovskite polymorphs present to be identified, and defined a wide region of coexistence of the orthorhombic and rhombohedral phases that spread between x = 0.4 and 0.9. Lattice parameters were determined for each member of the two perovskite solid solutions, isostructural with orthorhombic YMnO_3_ and rhombohedral BiFeO_3_, respectively. A gradual change of the lattice parameters with the composition was found, which relates not only with substitutions of ions with different ionic radii, but also with the progressive disappearance of the Jahn-Teller effect. Phase transitions were investigated by DTA and temperature-dependence X-ray diffraction, and the magnetic and electric ordering temperatures were determined for compositions from x = 0.9 to x = 1.

## Figures and Tables

**Table 1:** Reaction time, maximum temperature before decomposition, and lattice parameters (in Å) obtained using a least-square refinement method (Errors with 3*σ) (CELREF) [27].

x	Reaction Time (h)	Max. T (°C)	Orthorhombic Pnma			Rhombohedral R3c h	
			a	b	c	a	C
0	8	900	5.810(6)	7.37(1)	5.252(6)	--	--
0.1	7	700	5.76(1)	7.42(2)	5.26(2)	--	--
0.2	5	600	5.69(2)	7.46(3)	5.26(2)	--	--
0.3	4	550	5.66(2)	7.52(2)	5.27(1)	--	--
0.4	3	550	5.65(2)	7.58(2)	5.30(2)	--	--
0.5	5	550	5.621(3)	7.629(6)	5.332(3)	--	--
0.6	7	550	5.61(1)	7.69(2)	5.36(1)	5.54(6)	13.37(2)
0.7	9	600	5.59(1)	7.73(2)	5.41(1)	5.54(2)	13.44(1)
0.8	11	700	5.58(1)	7.78(1)	5.45(1)	5.56(2)	13.510(6)
0.9	20	750	5.59(1)	7.82(2)	5.51(2)	5,55(2)	13.761(6)
0.925	18	825	--	--	--	5.56(1)	13.79(1)
0.95	17	825	--	--	--	5.567(3)	13.820(6)
1	14	750	--	--	--	5.580(3)	13.87(1)

**Table 2:** Assignment of vibrational modes of BiFeO_3_ (in cm^−1^) [30].

Exp.	Ref. [30]	Exp.	Ref. [30]	Exp.	Ref. [30]	Exp.	Ref. [30]
E_g_ (TO) ^1^	E_g_ (TO) ^1^	E_g_ (LO) ^2^	E_g_ (LO) ^2^	A_1g_ (TO) ^1^	A_1g_ (TO) ^1^	A_1g_ (LO) ^2^	A_1g_ (LO) ^2^
72	74	79	81	143	149	-	178
137	132	174	175	221	223	-	229
240	240	-	242	310	310	-	502
263	265	294	276	542	557	-	591
278	278	-	346				
349	351	-	368				
373	374	-	430				
434	441	472	468				
521	523	622	616				

^1^ TO: Transversal Optic phonon frequencies; ^2^ LO: Longitudinal Optic phonon frequencies.

**Table 3:** Assignment of vibrational modes of YMnO_3_ (in cm^−1^) [31,32].

Symmetry	Exp.	Ref. [31]	Ref. [32]	Calc. (Ref. [32])
A_g_	149	151	150	165
A_g_	186	188	187	217
A_g_	284	288	289	265
A_g_	319	323	324	302
A_g_	395	396	398	373
A_g_	494	497	497	483
A_g_	515	518	519	498
B_2g_	149	151	152	179
B_2g_	219	220	222	234
B_2g_	319	317	318	297
B_2g_	338	341	342	351
B_2g_	483	481	481	497
B_2g_	533	537	539	523
B_2g_	616	616	618	618
B_1g_	-	205	205	226
B_1g_	-	284	285	269
B_1g_	384	383	382	327
B_1g_	-	-	-	414
B_1g_	-	-	-	647
B_3g_	-	178	179	184
B_3g_	-	336	337	332
B_3g_	-	-	-	410
B_3g_	-	-	-	491
B_3g_	-	-	-	642
	641	Defect origin		
